# High-Temperature Compressive Response of SiC_p_/6092Al Composites under a Wide Range of Strain Rates

**DOI:** 10.3390/ma14216244

**Published:** 2021-10-20

**Authors:** Yongyong Suo, Jintao Li, Zhilun Deng, Bo Wang, Quanzhao Wang, Dingrui Ni, Purong Jia, Tao Suo

**Affiliations:** 1 School of Mechanics, Civil Engineering and Architecture, Northwestern Polytechnical University, Xi’an 710129, China; yysuo09@mail.nwpu.edu.cn (Y.S.); prjia@nwpu.edu.cn (P.J.); 2 School of Aeronautics, Northwestern Polytechnical University, Xi’an 710072, China; lijintaogl@mail.nwpu.edu.cn (J.L.); dengzl0924@mail.nwpu.edu.cn (Z.D.); suotao@nwpu.edu.cn (T.S.); 3 Shaanxi Key Laboratory of Impact Dynamics and Engineering Application (IDEA), Northwestern Polytechnical University, Xi’an 710072, China; 4 Shi-Changxu Innovation Center for Advanced Materials, Institute of Metal Research, Chinese Academy of Sciences, 72 Wenhua Road, Shenyang 110016, China; drni@imr.ac.cn

**Keywords:** metal matrix composites, dynamic compressive properties, high temperature, strain-rate-dependent behavior, particle failure mode

## Abstract

The high-temperature dynamic compressive properties of a 30 vol.% SiC_p_/6092Al composite, fabricated using powder metallurgy, were experimentally investigated using the split Hopkinson pressure bar system with an electric furnace. Three different ambient temperatures, namely, room temperature, 200 °C, and 350 °C, were adopted, and the dynamic tests of the composite specimens were conducted at strain rates ranging from 1500 to 4500 s^−1^. The experimental results showed that the flow stress of the composite was generally insensitive to strain rates at room temperature. However, the composite started exhibiting different strain-rate-dependent behaviors as the temperature increased, and the flow stress nonlinearly varied with increasing temperature. In addition, the microscopic images of the specimens showed that the microscopic failure mechanisms of the composite were greatly influenced by the ambient temperature and strain rate. Specifically, the percentage of failed particles decreased with rising temperature and the dominating failure mode of particles changed significantly as the strain rate increased.

## 1. Introduction

Compared to pure metal and alloy materials, particle-reinforced metal matrix composites (PRMMCs) have been more widely used in aviation, aerospace, and military applications due to their many advantages, such as high specific strength, high specific stiffness, and improved high-temperature performance. Structural components with PRMMCs as the main material frequently encounter high-speed impacts by various foreign objects, as well as high-temperature conditions in their routine applications. For example, the metal matrix composite structures employed in aircraft engines and various missiles may be impacted by foreign objects in their high-temperature service environment, which is generally induced by frictional heating during operation. Over the past several decades, numerous achievements have been made regarding the quasi-static mechanical properties of PRMMCs [1,2,3,4,5], but studies on their dynamic mechanical properties are still insufficient, especially under high-temperature conditions. However, there is evidence [6,7,8,9,10,11,12,13,14,15] that the dynamic mechanical properties and failure mechanisms of PRMMCs under high-speed deformation are significantly different from those under quasi-static conditions. Therefore, it is important to study the mechanical behaviors of PRMMCs at high strain rates, especially under the coupling action of high temperatures and high strain rates.

Existing experimental studies on the dynamic mechanical behaviors of PRMMCs have mainly been conducted using the split Hopkinson pressure bar (SHPB) system. Most of these studies were focused on the effect of particle characteristics on the dynamic mechanical properties of PRMMCs at room temperature. San Marchi et al. [16] and Liu et al. [17] independently carried out dynamic compressive experiments on PRMMCs containing particles of various sizes, and their results revealed that particle size exerts a significant influence on the dynamic flow stress and strain rate sensitivity of composites, that is, the flow stress increases while the strain rate sensitivity decreases with decreasing particle size. Min Chul et al. [18] and Lee et al. [19] found out from dynamic compressive tests that the dynamic strength and fracture energy of multi-sized particle composites are generally higher than those of mono-sized composites. Lee et al. [20] concluded from another experimental investigation that both the compressive strength and failure strain of PRMMCs under dynamic loadings are higher than those under quasi-static conditions, and the main reasons are the strain-hardening rate and the matrix melting caused by adiabatic heating under dynamic loadings, respectively. An experimental investigation of the failure mechanisms of PRMMCs under dynamic loadings conducted by Zhang et al. [21] showed that the main factors influencing the damage and failure mechanisms of the composite mainly include particle size, distribution, interface properties, and the adiabatic heating effect. Zhang et al. [6] investigated how the fabrication route affects the dynamic behaviors of PRMMCs, and their results demonstrated that the fabrication route has a significant influence on the strain-hardening rate of PRMMCs. Another experimental study on the dynamic compressive behaviors of a 60 vol.% TiB_2_/Al composite and aluminum alloys conducted by Zhu et al. [8] indicated that the pure matrix material barely exhibited any strain rate effect, while composites with particles generally showed obvious strain rate sensitivity, and they also found that adiabatic heating could not only accelerate the softening and flow behaviors of the matrix but also hinder the low strain failure behavior of the composite. Owalabi et al. [22] investigated the effects of particle addition on the strain localization induced by adiabatic heating under dynamic loading conditions. Their findings showed that although particle addition improves the strength and stiffness of composites, it usually leads to strain localization and adiabatic shear failure in the matrix. This study also suggested that the strain localization along the shear band induced by adiabatic heating generally results in the catastrophic failure of composites in the final stage of deformation.

Through the above literature review, it can be seen that most existing studies on the dynamic mechanical behaviors of PRMMCs were carried out at room temperature, and research conducted at high temperatures has rarely been reported. Independent studies conducted by Tang et al. [23] and Wang et al. [24] on the compressive behaviors of PRMMCs at different temperatures and strain rates (0.001–1 s^−1^) showed that the flow stress decreases with increasing ambient temperature but increases as the strain rate increases. Similar conclusions can be drawn from high-temperature compressive tests conducted on a 15 vol.% SiC/8009Al composite in the same strain range as the two previous studies carried out by Chen et al. [25]. Their experimental results also showed that the flow stress initially increased with increasing deformation at high temperatures, and after reaching the peak, it started to slightly decline and gradually stabilizes at a certain level. Wang et al. [26] studied the torsional deformation behaviors of PRMMCs at different temperatures (400–500 °C) and strain rates (0.001–10 s^−1^), and their experimental results also demonstrated that the shear flow stress increased with increasing strain rate and decreasing temperature. Another study conducted by Sun et al. [27] investigated the effect of ambient temperatures on the flow stress of SiC_p_/Al composites at a strain rate of 2000 s^−1^. Their conclusion on the variations in flow stress with temperature was similar to that of the aforementioned studies. However, the authors of this work failed to consider the impact of ambient temperatures on the strain rate sensitivity and microscopic failure mechanisms of PRMMCs.

Considering the abovementioned investigations in the relevant literature, it is clear that existing studies on the high-temperature mechanical properties of PRMMCs were mainly conducted at low strain rates that are much closer to quasi-static deformation, while reports regarding the coupling effect of high temperatures and high strain rates on the mechanical properties of PRMMCs are currently very rare. However, in practical applications, such as high-speed flying missiles, many composite structures not only induce high temperature by friction with the air but also are impacted by foreign objects at high speeds. Therefore, it is necessary to study the mechanical behaviors of PRMMCs under the coupling action of high temperatures and high strain rates. In this work, a 30 vol.% SiC_p_/6092Al composite was first prepared using powder metallurgy, and then a series of dynamic compressive experiments were carried out at different ambient temperatures using a split Hopkinson pressure bar system with an electric furnace. Finally, the mechanical behaviors and microscopic failure mechanisms of the composite under the coupling action of high temperatures and high strain rates were extensively studied.

## 2. Materials and Experiments

### 2.1. Material Fabrication

**Figure 1:**
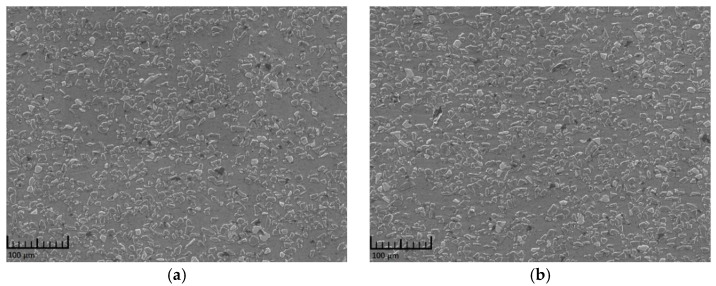
Particle distribution in the as-prepared PRMMCs. (**a**) SEM image of the cross section; (**b**) SEM image of the longitudinal section.

The constituent materials of the composite employed in this work were a 6092 aluminum alloy and SiC particles (CHAOWEI-NANO Co., Ltd, Shanghai, China) with an average size of 14 μm. The SiC particles and alloy powders were mixed for 8 h in a bi-axis rotary mixer (DROIDE Co., Ltd., Beijing, China) with a rotation speed of 500 rpm and a ball-to-powder ratio of 1:1. The as-mixed powders were cold-pressed in a cylindrical die under a pressure of 50 MPa and then hot-pressed under a pressure of 100 MPa at 600 °C for 30 min. To reduce the porosity of the material and obtain a high-density composite, the ingot was then subjected to hot extrusion process at 460 °C with an extrusion ratio of 10:1. After this treatment, the relative density of the composite ingot reached as high as 99.8%. The as-prepared ingot was then machined into cylindrical specimens (the cylinder axis parallel to the extrusion axis) with a diameter and length of 4 mm using wire cutting technology. The prepared specimens were all polished, kept at 540 °C for 2 h, water quenched, and finally aged at 175 °C for 6 h. Following these processes, the specimens were used to conduct the dynamic compressive tests. The longitudinal (along the extrusive axis) and cross (vertical to the extrusive axis) sections of a specimen were carefully polished to examine particle distributions. As shown in the micrograph in Figure 1, a relatively uniform distribution of particles in the composite with hardly any clusters was observed. In addition, no preferred particle orientation was found along the extrusion axis.

### 2.2. Experimental Procedures

**Figure 2:**
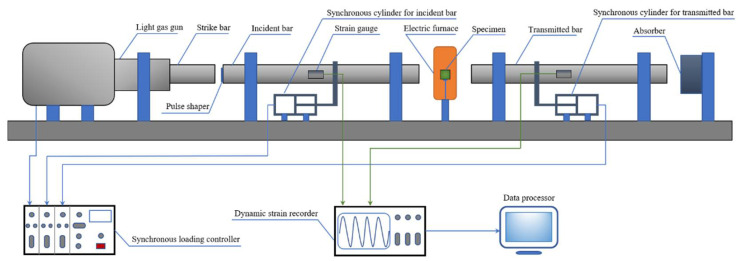
Diagram of the SHPB system used in this work.

**Figure 3:**
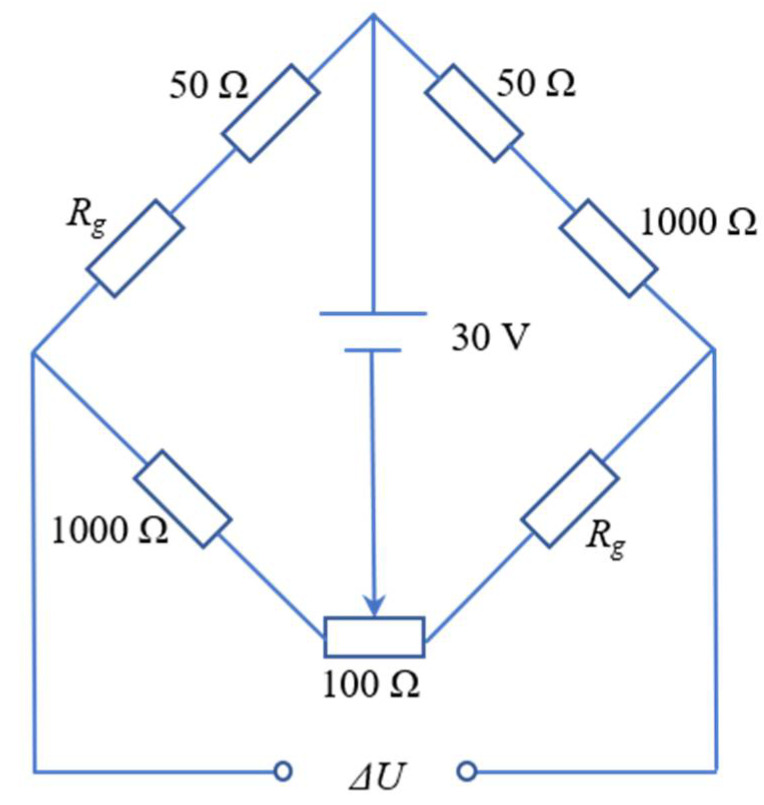
Wheatstone bridge adopted in the test.

In order to investigate the dynamic mechanical behaviors of the SiC_p_/6092Al composite under the coupling action of temperatures and strain rates, a series of dynamic impact tests on the composite were carried out using a split Hopkinson pressure bar system with an electric furnace. Three different temperatures were utilized in the tests, namely, room temperature (RT), 200 °C, and 350 °C. During the high-temperature tests, in order to minimize the influence of high temperature on the modulus of the incident and transmitted bars, which would have made the experimental results inaccurate, a self-designed high-temperature synchronous loading device (HTSLD) was employed to modify the traditional Hopkinson pressure bar system, as shown in Figure 2. With the aid of this device, specimens could be independently heated without overheating the bars. Thus, the influence of high temperature on the modulus of the bars and the final results was eliminated to the greatest possible extent. The working principle of HTSLD can be described as follows: when specimens were heated in the furnace, the incident and transmitted bars were kept outside the furnace by the synchronous cylinders installed on each bar to avoid heating. Upon the full heating of the specimens, the synchronous cylinders simultaneously pushed the two bars into the furnace to contact with the heated specimen. Then, the synchronizer controlled the striker to impact the incident bar at a high speed to complete the high-temperature impact loading. It should be noted that the heating rate in tests remained at 10 °C/min to minimize the effect of thermal shock. Additionally, the experiments were carried out only if the specimens were maintained at the target temperature for at least 15 min for uniform-heating purposes. The critical parameters of the striker, incident, and the transmitted bars were identical and listed as follows: the modulus was 184.95 GPa, the diameter was 18.9 mm, and the wave speed in the bars was 4850 m/s. Two groups of strain gauges (*R_g_* = 1000 Ω) were attached to the incident and transmitted bars. During the tests, a high-speed dynamic signal acquisition system was employed to record the output voltage signals of the two channels, and the sampling frequency was 10 MHz. For each channel, the two strain gauges were connected to the opposite legs of the bridge (half bridge configuration), as shown in Figure 3. The output voltage signals were converted into strain value using Equation (1), where *k* refers to the gage factor of the strain gage and Δ*U* is the output voltage. Then, based on the one-dimensional stress wave theory, the strain, strain rate, and stress of the specimen were calculated from the strain in the two bars according to Equation (2), where *C_B_* is the wave speed in the bars, *L_S_* is the length of the specimen, *E_B_* is the modulus of the bars, and *A_B_* and *A_S_* are the cross-sectional areas of the bars and the specimen, respectively. Additionally, *ε_R_* and *ε_T_* reflect the strain amplitudes of the reflected and transmitted waves, respectively. It should be pointed out that in this work, repetitive experiments were carried out for each working condition to ensure the repeatability of the experimental results. To ensure the alignment condition of the SHPB system, a calibration to the system was performed before the test by launching the striker directly on the incident bar. The calibration results showed that the incident wave perfectly coincided with the transmitted wave without any reflected signal due to the two bars sharing the same size, material, and impedance. This indicated that the alignment and contact conditions of the SHPB system perfectly met the requirements for the impact tests.

(1) $$\varepsilon =\frac{2100\cdot \Delta U}{1000\cdot k\cdot \left(30-\Delta U\right)}$$

(2) $$\{\begin{array}{l} \dot{\varepsilon }=-2\frac{C_{B}}{L_{S}}\varepsilon_{R}\\ \varepsilon =-2\frac{C_{B}}{L_{S}}\int_{0}^{t}\varepsilon_{R}dt\\ \sigma =\frac{A_{B}}{A_{S}}E_{B}\varepsilon_{T} \end{array}$$

**Figure 4:**
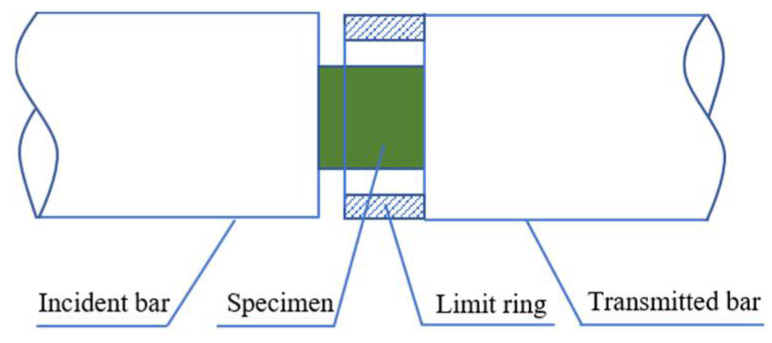
Diagram of the limit ring.

Additionally, in order to examine the effect of strain rates on the microscopic failure mechanisms of the composite, it was necessary to ensure that the specimens experienced the same deformation at various strain rates. Therefore, several limit rings were designed according to the sizes of the specimen and the bars to ensure that the specimen produced the required deformation during the tests at different strain rates, as shown in Figure 4.

## 3. Results and Discussion

### 3.1. Analysis of Stress Equilibrium and Strain Rate History

**Figure 5:**
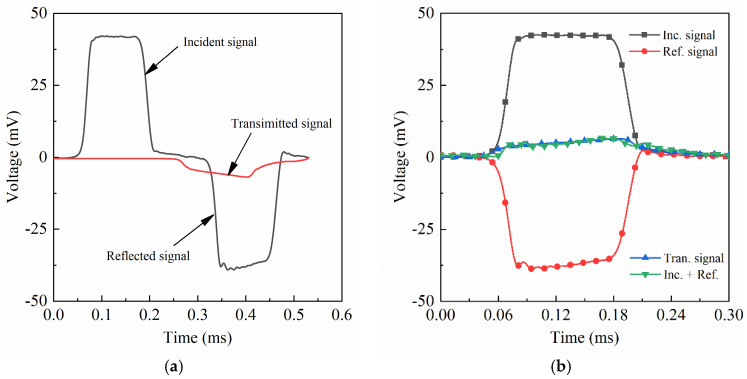
Stress waves and the stress equilibrium result of a typical test. (**a**) Signals of the stress waves in a test; (**b**) results of the stress equilibrium.

Figure 5a shows the voltage signals of the incident, reflected, and transmitted waves in a typical dynamic test (~4300 s^−1^) at room temperature. It must be pointed out is that in order for the specimens to produce uniform deformation at a constant strain rate, the incident pulse needed to be shaped by placing an appropriate amount of plasticine on the impact end of the incident bar in all of the tests in this work. The amount of plasticine was dependent on the required strain rates and temperatures. According to the one-dimensional stress wave theory, the accuracy of experimental results is greatly influenced by the stress equilibrium condition of the test piece in dynamic experiments. The stress equilibrium results of the specimen in Figure 5a are depicted in Figure 5b, which indicates that the equilibrium results fit well with Equation (3), where *ε_I_* represents the strain amplitude of the incident wave. Thus, the accuracy of the test results can be safely guaranteed.

(3) $$\varepsilon_{I}+\varepsilon_{R}=\varepsilon_{T}$$

**Figure 6:**
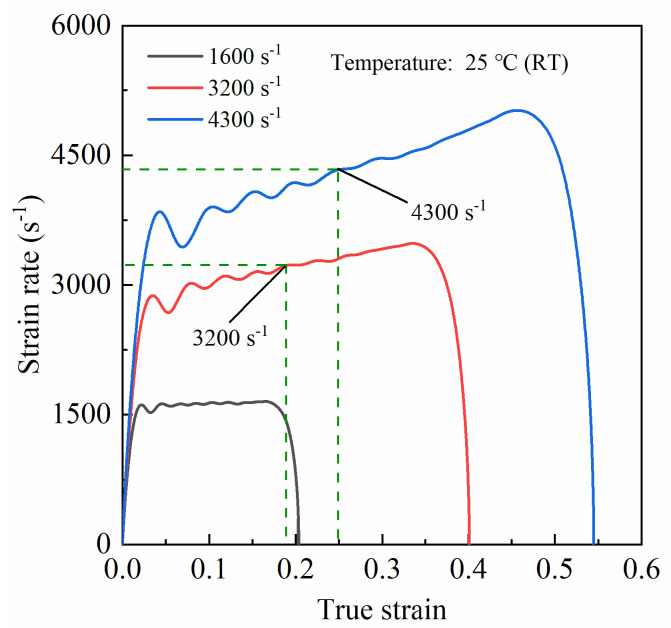
Strain rate history curves under various impact speeds.

During a typical SHPB test, the striker is launched by the sudden release of a light gas and accelerates until it impacts the incident bar at a certain speed. Theoretically, the specimen will deform at a uniform strain rate as long as the striker impacts the incident bar at a constant speed, that is, a plateau should be present on the strain rate history curve, and during this plateau period, the strain rate is almost constant. However, in the experiments of this work, it was found that not all of the strain rate history curves were consistent with the theoretical situation. Specifically, at lower-speed deformation (~1600 s^−1^), the strain rate history of the specimen generally conformed to the theoretical situation and could reach a very stable platform stage (as shown in Figure 6). However, for higher-speed deformations (>3000 s^−1^), the strain rate of the specimens continued to slowly increase after rapidly rising to a certain level rather than maintaining a constant value (as shown in Figure 6). It should be noted that only the curves at room temperature are presented in Figure 6, as the results for higher temperature conditions showed the same trend as that at room temperature. The main reasons for this phenomenon can be explained as follows. At lower-speed deformation, the plastic deformation during compression for the same period of time was relatively small, as was the heat converted from plastic work. Thus, the adiabatic heating exerted little influence on the softening behavior of the matrix, which led to a relatively stable strain rate level in the specimen. However, at higher-speed deformation, the specimens generally underwent serious plastic deformation in a very short period of time, which caused a sharp rise in the temperature of the specimen due to adiabatic heating. Thus, the softening behavior of the aluminum alloy matrix was accelerated, driving the strain rate of the specimen to continuously increase. For the ultra-high strain rate, the increasing trend of strain rate with deformation became more significant because of the great influence of adiabatic heating on the matrix. However, given that the strain rate continued to increase approximately linearly with deformation at the plateau stage, the middle value of this stage can be considered to be the average strain rate of the specimen under high-speed deformation, as shown in Figure 6.

### 3.2. Influence of Temperature on the Stress–Strain Curves

**Figure 7:**
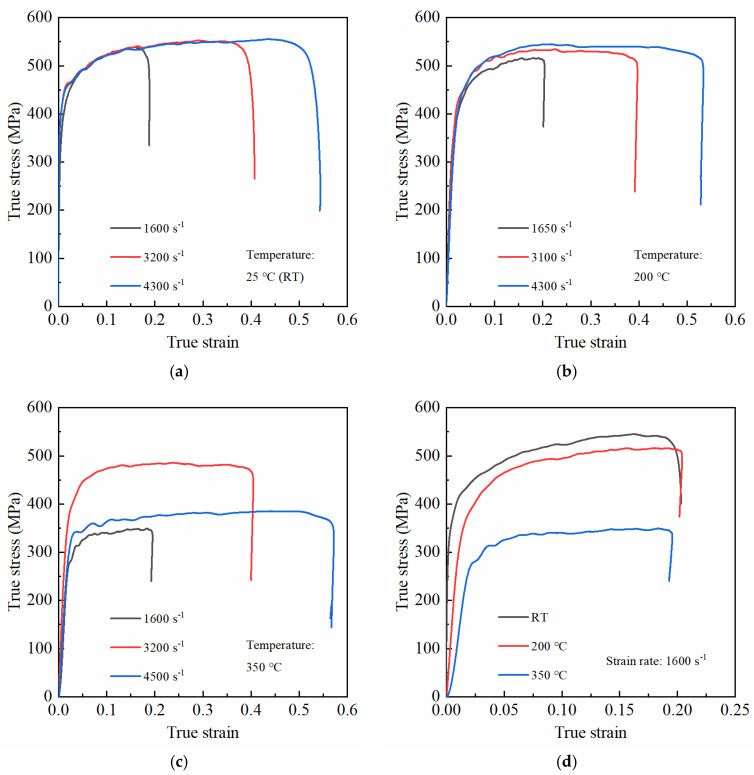
Influence of the strain rate and temperature on the stress–strain curves of PRMMCs. (**a**) True stress–strain curves at RT; (**b**) true stress–strain curves at 200 °C; (**c**) true stress–strain curves at 350 °C; (**d**) true stress–strain curves at 1600 s^−1^.

In this section, the dynamic mechanical behaviors of PRMMCs were experimentally investigated under various temperature conditions (RT, 200 °C, and 350 °C), and the experimental results are shown in Figure 7. It is noteworthy that tests under each loading condition were repeated at least three times for reproducible results; however, only one curve for each case is displayed in Figure 7 for the sake of clarity and simplicity. Additionally, it is obvious in the graph that the strain-rate-dependent behaviors of the SiC_p_/6092Al composite greatly varied with the ambient temperature.

The dynamic compressive responses of the composite at room temperature are shown in Figure 7a. As is clearly shown in the graph, the yield stress of the composite at 1600 s^−1^ was lower than that at 3200 s^−1^. However, as the strain rate increased from 3200 to 4300 s^−1^, there were no more apparent increases in the yield stress of the composite, which suggested that the yield stress of the SiC_p_/6092Al composite could only exhibit a strain-rate-strengthening effect within a certain strain rate range at room temperature. On the other hand, the flow stress after yielding was almost unchanged for each strain rate level at the same deformation. Thus, it is reasonable to consider that the flow stress of the SiC_p_/6092Al composite had no strain rate effect at room temperature. This is mainly because the flow stress of most aluminum alloys (including 6092Al) is generally rate-independent at room temperature [28,29,30]; thus, the flow stress of PRMMCs with aluminum alloys as their matrix is normally insensitive to strain rates. Additionally, a further investigation of the curves indicated that the composite exhibited an obvious strain-hardening behavior during the early stages after yielding. However, as deformation increased, the strain-hardening rate gradually decreased, and this trend was independent of the strain rate. There are two reasons for this phenomenon: first, although one part of the heat converted from the plastic work during loading immediately dissipated, the other part could still increase the internal temperature of the specimen, which softened the matrix to a certain extent; second, particles in the composite started to fracture as the loading continued, which weakened the strengthening effect of particles [21,31,32]. Furthermore, the curves in Figure 7a also demonstrate that the flow stress continued to increase with deformation although the strain-hardening rate of the composite decreased as the loading continued, which indicated that the thermal softening of the matrix and the failure of particles could only partially rather than completely counteract the strain-hardening effect at room temperature. Thus, from a macroscopic point of view, the flow stress of the composite still showed an increasing trend with increasing deformation. Finally, it should be noted that the strain-hardening rate of the composite was greater at a lower strain rate and the strain-hardening effect weakened as the strain rate increased, mainly because the adiabatic heating was generally more significant at higher strain rates than that at lower strain rates.

Figure 7b shows the dynamic compressive responses of the composite at 200 °C. As is shown in the graph, the yield stress improved as the strain rate increased at 200 °C, which was similar to that at room temperature. On the other hand, unlike that at room temperature, the flow stress of the composite at 200 °C increased with increasing strain rate and the composite started to exhibit a positive rate-dependent behavior, which suggested that the ambient temperature exerted an influence on the strain-rate-dependent behavior of the composite. This is mainly because most aluminum alloys exhibit different rate-dependent behaviors with increasing ambient temperature, although they are generally insensitive to strain rates at room temperature [29]. In addition, unlike the observation made at room temperature, the strain-hardening rate of the composite at a higher strain rate was obviously higher than that at a lower strain rate during the initial stage after yielding at 200 °C, indicating that the strain-hardening behavior of the composite was also influenced by the ambient temperature. However, as the deformation increased, the strain-hardening rate of the composite gradually decreased, which indicated that with an increase in plastic deformation, the softening behavior induced by adiabatic heating and particle failure started to counteract the strain-hardening effect. Additionally, as the deformation continued to increase, a stable plateau occurred in the stress–strain curve, suggesting that the strain-hardening effect of the composite was completely counteracted by the softening that occurred in this stage.

The dynamic compressive responses of the composite at 350 °C are shown in Figure 7c. It can be seen from the graph that with a further increase in temperature, the strain rate sensitivity of the composite showed a significantly different pattern than that at room temperature and 200 °C. First, the strain rate sensitivity of the composite rapidly increased as the temperature increased from 200 to 350 °C. Specifically, the increase in the flow stress at 350 °C was obviously greater than that at 200 °C under the same deformation when the strain rate increased from 1600 to 3200 s^−1^. Second, the strain rate sensitivity of the composite non-monotonically varied when the temperature rose to 350 °C, that is, although the flow stress of the composite at 3200 s^−1^ was significantly higher than that at 1600 s^−1^, it dramatically decreased as the strain rate increased to 4500 s^−1^. The main reasons for this can be summarized as follows: on one hand, although the flow stress was expected to increase as the strain rate increased, the softening behavior of the aluminum alloy matrix was simultaneously promoted by the high temperature; on the other hand, the adiabatic heating during loading was more significant at higher strain rates, which made the temperature drastically rise in the composite, thus promoting its softening behavior. Therefore, from a macroscopic point of view, the flow stress of the composite at 4500 s^−1^ was much lower than that at 3200 s^−1^. For comparison, the flow stress at 15% applied strain for each loading case is summarized in Table 1.

The stress–strain curves of the composite at 1600 s^−1^ under various temperature conditions are depicted in Figure 7d. As is clearly shown, the modulus, yield stress, flow stress, and strain-hardening rate of the composite decreased as the temperature increased from RT to 350 °C. Furthermore, the flow stress nonlinearly varied with ambient temperature. In more detail, the flow stress only slightly decreased as the temperature increased from RT to 200 °C; however, when the temperature continuously increased to 350 °C, the flow stress tended to drastically decrease. This indicated that there may have been a critical transition temperature for the flow stress of the composite. When the ambient temperature did not exceed this critical temperature, the variation in flow stress with temperature was relatively small, but when it exceeded the critical temperature, the flow stress began to sharply decrease with rising temperature.

### 3.3. Dynamic Compressive Deformation and Failure Mechanisms

#### 3.3.1. Specimen Morphology after Compressive Tests

**Figure 8:**
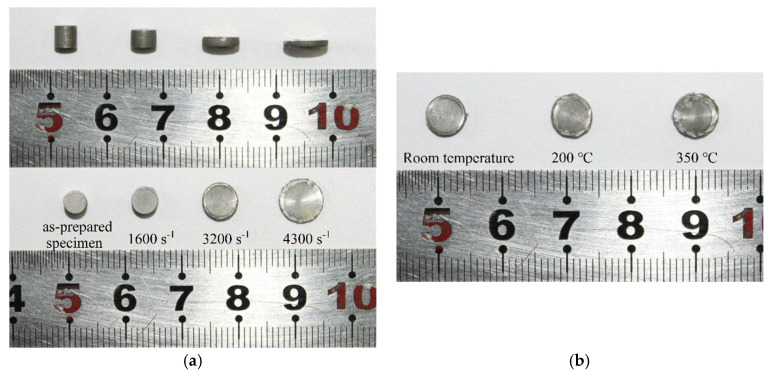
Morphology of the specimens after tests. (**a**) Tests at room temperature; (**b**) tests at the strain rate of 3200 s^−1^.

The specimens after compressive tests at various strain rates under room temperature are shown in Figure 8a. As can be seen from the graph, as the strain rate increased, the specimens only showed a decrease in the longitudinal dimension (along the loading direction) and an increase in the transverse dimension (vertical to the loading direction) but maintained their integrity after the tests. Additionally, as the impact speed (i.e., strain rate) increased, none of the specimens broke into pieces and no large penetrating cracks were observed on the specimens. However, a further examination revealed that some surface cracks, which increased in number with increasing strain rate, appeared on the lateral surface of the specimens, and most of these cracks were parallel and distributed 45 degrees from the loading direction. Compared with the experimental results listed in [7,20], where specimens generally failed in the shear fracture mode at about 45 degrees from the loading direction, the specimens in this study still maintained good integrity after loading at various strain rates, though their sizes dramatically changed. This indicates that the SiC_p_/6092Al composite fabricated in this study exhibited good toughness because of the excellent ductility of its matrix, i.e., the 6092 aluminum alloy. Thus, compared with the 6092Al, the fabricated SiC_p_/6092Al composite not only showed improved strength but also maintained fairly good toughness.

The specimens after compressive tests at 3200 s^−1^ under various ambient temperature conditions are shown in Figure 8b. As is shown in the image, the transverse dimension of the specimens after impact slightly increased as the temperature increased. Moreover, no cracks were observed on the lateral surface of the specimens at room temperature; however, as the temperature rose, cracks started to appear on the lateral surface and tended to propagate from the outer edge to the interior of the specimens. This phenomenon implied that temperature barely affected the macroscopic ductility of the composite, but it did affect the crack initiation and propagation behaviors of the composite. The main reason for this might be that the high temperature intensified the influence of the mismatch in the thermal expansion coefficients of the matrix and particles, which promoted crack initiation at the outer edge of the specimens.

#### 3.3.2. Microscopic Failure Mechanisms

**Figure 9:**
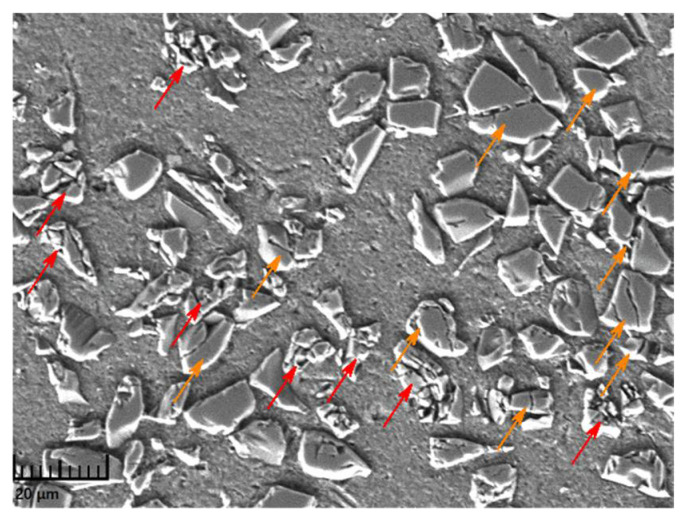
Particle failure mode in a specimen (yellow arrows: cracked particles; red arrows: smashed particles).

Through an investigation of the micromorphology of the longitudinal section of each specimen after tests, it was found that the failure mode in the specimens was influenced by the ambient temperature, the applied strain, and the strain rate during loading. In the experiments of this study, the dominating failure mode in the composite was particle fracture, and the failed particles could be divided into two types: cracked (yellow arrows) and smashed (red arrows) particles, as shown in Figure 9. The cracked particles were mainly characterized by several large penetrating cracks that broke the particles into a few large pieces. For the smashed particles, many secondary cracks evolved from the main penetrating cracks, which then destroyed the particles into a large number of fine debris.

**Figure 10:**
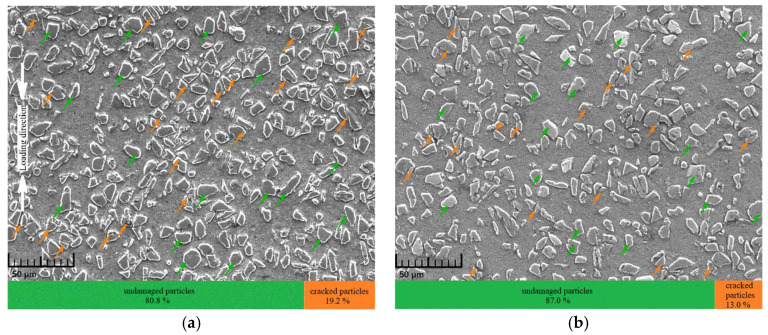
Micromorphology of specimens after 20% applied strain at 1600 s^−1^. (**a**) SEM graph of the specimen at RT; (**b**) SEM graph of the specimen at 350 °C.

In order to investigate the effect of ambient temperature on the microscopic failure characteristics in the composite, SEM images of the longitudinal sections of specimens after experiments (applied strain = 20%; strain rate = 1600 s^−1^) at different temperatures were extensively studied. It should be pointed out that the stacked bar charts at the bottom of each figure were calculated based on the average values of at least three different regions on the longitudinal section of each specimen. As is clearly shown in Figure 10, the ambient temperature had a remarkable influence on the failure behavior of particles in the composite, that is, the fraction of failed particles decreased as the ambient temperature rose. Specifically, at room temperature, there were about 19.2% failed particles, most of which were directly cracked, with only a few smashed into fine debris. However, as the temperature rose to 350 °C, the fraction of failed particles decreased to about 13.0%, all of which were directly cracked with hardly any smashed particles. The main reason can be summarized as follows: the matrix gradually softened as the ambient temperature rose, so the constraint of the matrix to particles and the ability of the matrix to transfer loads to particles started to weaken. Therefore, the stress in particles decreased with rising temperature, leading to a lower particle breakage probability at high temperatures. It can also be seen through a further examination of the SEM images that although the fraction of failed particles varied with the ambient temperature, interfacial debonding between particles and the matrix was seldomly observed, which suggested that the SiC_p_/6092Al composite fabricated in this study had relatively strong interfaces between the matrix and particles.

**Figure 11:**
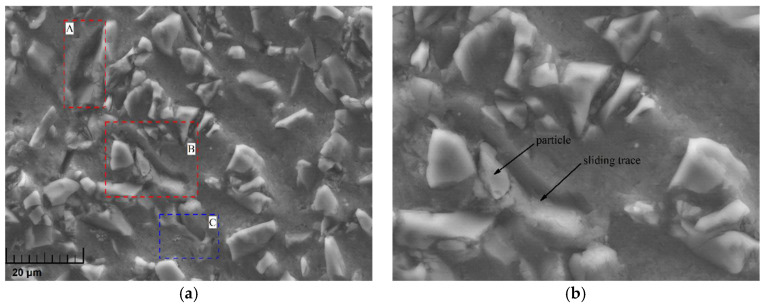
Local micromorphology of a specimen at 1600 s^−1^ under the 350 °C condition. (**a**) Morphological characteristics: particle sliding (A, B) and matrix flowing (C); (**b**) detailed morphology in region B.

The ambient temperature not only influenced the fracture behavior of particles but also exerted a great impact on the flow/softening behavior of the metal matrix. When the specimen was impacted at room temperature, one part of the heat generated from the plastic work was rapidly dissipated due to the low ambient temperature while the other part of the heat increased the temperature of the specimen, which merely softened the matrix to a small extent that was not enough to produce obvious plastic flow in the matrix. However, as the ambient temperature rose to 350 °C, the dissipated heat was greatly reduced, making the temperature of the specimen sharply rise, which accelerated the softening of the matrix. The micrograph of a specimen impacted at 1600 s^−1^ under the 350 °C condition, as shown in Figure 11, indicates that particles could easily slide inside the greatly softened matrix during loading, leaving deep sliding traces behind them.

**Figure 12:**
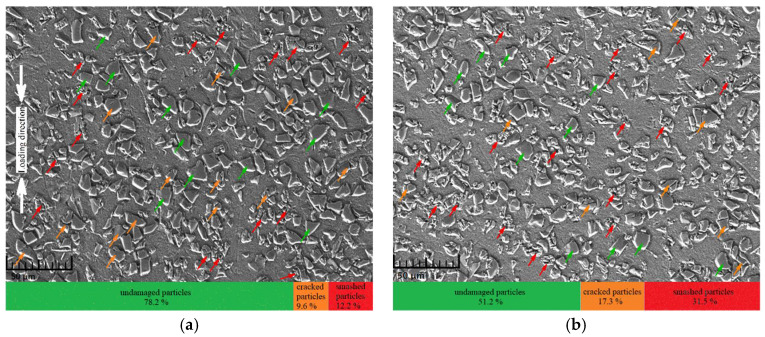
Micromorphology of specimens at 4500 s^−1^ under the RT condition. (**a**) SEM graph of the specimen under 20% deformation; (**b**) SEM graph of the specimen under 50% deformation.

To study the influence of deformation on the microscopic failure mechanisms in the composite, SEM images of the longitudinal section of specimens after experiments (~4500 s^−1^) at different applied strains under the room temperature condition were extensively investigated. The results are shown in Figure 12, from which it can be seen that particles in the two cases showed varying degrees of damage. Specifically, the fraction of failed particles at an applied strain of 20% was about 21.8%, and it increased rapidly to about 48.8% as the applied strain increased to 50%, indicating that the probability of particle failure tended to increase with increasing applied strain, which is consistent with previously reported experimental results [21,32]. In addition, through a closer examination of Figure 10a and Figure 12a, an important conclusion that the strain rate exerted a remarkable influence on the failure mode of particles could be drawn. More specifically, almost all the failed particles were directly cracked (without any smashed particles) at the strain rate of 1600 s^−1^, as shown in Figure 10a. However, as the strain rate increased to 4500 s^−1^, most of the failure particles were smashed into fine debris (about 56% of all failed particles, as shown in Figure 12a) at the same deformation of 20% as that in the case of 1600 s^−1^. Additionally, with the deformation increasing to 50%, the ratio of smashed particles in all the failed ones correspondingly rose to 65%, as shown in Figure 12b. This indicated that although the percentage of all the failed particles was nearly independent of the strain rate (~19.2% at 1600 s^−1^ and ~21.8% at 4500 s^−1^), it could dramatically change the failure mode of particles in the composite; that is, the higher the strain rate was, the more likely the particles were to smash into fine debris. A potential reason for this is explained as follows: during the loading at high strain rates, the stress wave reverberated violently in the specimen, which repeatedly exposed the previously cracked particles to loadings by the stress wave, thus driving them to break into a large amount of fine debris.

**Figure 13:**
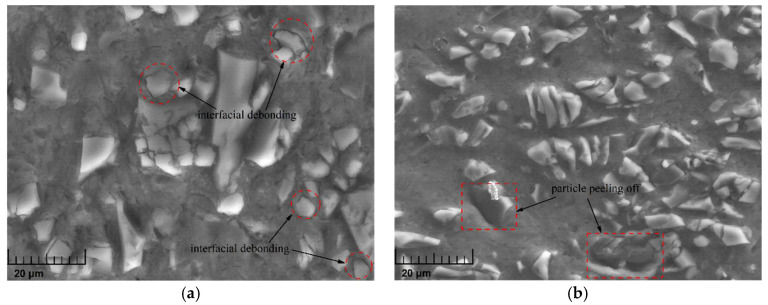
Detailed micromorphology of specimens at 4500 s^−1^ under the RT condition. (**a**) 20% applied strain; (**b**) 50% applied strain.

Existing studies have shown that the applied strain exerts great influence on the fracture behavior of particles; however, from the experimental results of this study, it can be concluded that the interfacial debonding behavior is also influenced by applied strain. The local morphological images of the two loading cases in Figure 12 are shown in Figure 13. In the case of 20% applied strain at 4500 s^−1^, Figure 13a shows that particles slightly debonding from the matrix could be frequently observed. However, in the case of 50% applied strain at 4500 s^−1^, the degree of interfacial debonding was greatly improved, so that particles could easily peel off the matrix and only leave voids behind them, as shown in Figure 13b. Additionally, a comparison between Figure 10a and Figure 13a indicates that interfacial debonding was more likely to occur at high strain rates due to the violently reverberating stress waves, although the specimens underwent identical deformation in the two cases.

Some important aspects of the above analysis should be emphasized. First, the ambient temperature exerted a significant influence on the mechanical behaviors of the matrix and particles, i.e., as the temperature rose, the degree of matrix softening increased, which made the particles less likely to fracture. Second, the probability of particle fracture was directly influenced by the applied strain subjected to the specimen, which means that the percentage of failed particles in the composites tended to rise as the applied strain increased. Lastly, the failure mode of particles was greatly affected by the strain rate, that is, the dominating failure mode of particles gradually changed from cracking to smashing as the strain rate increased.

## 4. Conclusions

In this work, an SiC_p_/6092Al composite was fabricated using SiC particles and 6092 aluminum alloys via the powder metallurgy method. The relative density of the composite was as high as 99.8%, and SEM graphs showed that particles in the composites were uniformly distributed. Then, an SHPB system with an electric furnace and a set of synchronous loading device was employed to carry out impact tests on the composite specimens at various ambient temperatures and strain rates. The experimental results showed that the dynamic compressive behaviors and the microscopic failure mechanisms of the composite were significantly influenced by the ambient temperature and the strain rate. The main conclusions of this study are listed as follows:

(1) The SiC_p_/6092Al composite was generally insensitive to strain rates at room temperature. However, as the temperature rose to 200 °C, the composite started to exhibit a positive strain-rate-dependent behavior, i.e., the flow stress gradually increased with increasing strain rate. When the temperature reached 350 °C, the strain rate sensitivity of the composite was further improved with a non-monotonical changing trend.
(2) The modulus, yield stress, flow stress, and strain-hardening rate of the SiC_p_/6092Al composite decreased as the ambient temperature rose, and the flow stress nonlinearly varied with the ambient temperature.
(3) Compared with 6092Al, the SiC_p_/6092Al composite fabricated in this work not only showed improved strength but also maintained good toughness.
(4) When subjected to impact loading, the dominating failure mode in the SiC_p_/6092Al composite was characterized by particle failure. The percentage of failed particles tended to decrease as the ambient temperature increased. Furthermore, the failure mode of particles was greatly influenced by the strain rate, that is, particles generally broke apart into large pieces at lower strain rates and they smashed into fine debris at higher strain rates.

## Figures and Tables

**Table 1:** The flow stress at 15% applied strain for each loading case.

Flow Stress (MPa)	Room Temperature (RT)	200 °C	350 °C
~1600 s^−1^	535.87	513.39	346.04
~3200 s^−1^	537.39	529.10	477.71
~4300 s^−1^	536.95	533.83	366.05

## Data Availability

The raw/processed data required to reproduce these findings cannot be shared at present since the data also form part of an ongoing study.

## References

[1] Meng Q., Wang Z. (2015). Prediction of interfacial strength and failure mechanisms in particle-reinforced metal-matrix composites based on a micromechanical model. Eng. Fract. Mech..

[2] Trofimov A., Drach B., Sevostianov I. (2017). Effective elastic properties of composites with particles of polyhedral shapes. Int. J. Solids Struct..

[3] Su Y., Ouyang Q., Zhang W., Li Z., Guo Q., Fan G., Zhang D. (2014). Composite structure modeling and mechanical behavior of particle reinforced metal matrix composites. Mater. Sci. Eng. A.

[4] Liu Q., Qi F., Wang Q., Ding H., Chu K., Liu Y., Li C. (2018). The influence of particles size and its distribution on the degree of stress concentration in particulate reinforced metal matrix composites. Mater. Sci. Eng. A.

[5] Zhang J., Ouyang Q., Guo Q., Li Z., Fan G., Su Y., Jiang L., Lavernia E.J., Schoenung J., Zhang D. (2016). 3D Microstructure-based finite element modeling of deformation and fracture of SiCp/Al composites. Compos. Sci. Technol..

[6] Zhang H., Ramesh K.T., Chin E.S.C. (2004). High strain rate response of aluminum 6092/B4C composites. Mater. Sci. Eng. A.

[7] Tan Z., Pang B., Qin D., Shi J., Gai B. (2007). The compressive properties of 2024Al matrix composites reinforced with high content SiC particles at various strain rates. Mater. Sci. Eng. A.

[8] Zhu D., Wu G., Chen G., Zhang Q. (2008). Dynamic deformation behavior of a high reinforcement content TiB2/Al composite at high strain rates. Mater. Sci. Eng. A.

[9] Tan Z., Pang B., Gai B., Wu G., Jia B. (2007). The dynamic mechanical response of SiC particulate reinforced 2024 aluminum matrix composites. Mater. Lett..

[10] Zhang H., Ramesh K.T., Chin E.S.C. (2005). Effects of interfacial debonding on the rate-dependent response of metal matrix composites. Acta Metall. Mater..

[11] Li Y., Ramesh K.T., Chin E.S.C. (2004). Comparison of the plastic deformation and failure of A359/SiC and 6061-T6/Al_2_O_3_ metal matrix composites under dynamic tension. Mater. Sci. Eng. A.

[12] Li Y., Ramesh K.T. (1998). Influence of particle volume fraction, shape, and aspect ratio on the behavior of particle-reinforced metal–matrix composites at high rates of strain. Acta Metall. Mater..

[13] Bao G., Lin Z. (1996). High strain rate deformation in particle reinforced metal matrix composites. Acta Metall. Mater..

[14] Yadav S., Chichili D.R., Ramesh K.T. (1995). The mechanical response of a 6061-T6 Al/Al_2_O_3_ metal matrix composite at high rates of deformation. Acta Metall. Mater..

[15] Chichili D.R., Ramesh K.T. (1995). Dynamic failure mechanisms in a 6061-T6 Al-Al_2_O_3_ metal-matrix composite. Int. J. Solids Struct..

[16] San Marchi C., Cao F., Kouzeli M., Mortensen A. (2002). Quasistatic and dynamic compression of aluminum-oxide particle reinforced pure aluminum. Mater. Sci. Eng. A.

[17] Liu J., Huang X., Zhao K., Zhu Z., Zhu X., An L. (2019). Effect of reinforcement particle size on quasistatic and dynamic mechanical properties of Al-Al2O3 composites. J. Alloy. Compd..

[18] Jo M.C., Choi J.H., Yoo J., Lee D., Shin S., Jo I., Lee S.-K., Lee S. (2019). Novel dynamic compressive and ballistic properties in 7075-T6 Al-matrix hybrid composite reinforced with SiC and B_4_C particulates. Compos. Part B Eng..

[19] Lee H., Choi J.H., Jo M.C., Lee D., Shin S., Jo I., Lee S.-K., Lee S. (2018). Effects of SiC particulate size on dynamic compressive properties in 7075-T6 Al-SiCp composites. Mater. Sci. Eng. A.

[20] Lee H., Sohn S.S., Jeon C., Jo I., Lee S.-K., Lee S. (2017). Dynamic compressive deformation behavior of SiC-particulate-reinforced A356 Al alloy matrix composites fabricated by liquid pressing process. Mater. Sci. Eng. A..

[21] Zhang J., Shi H., Cai M., Liu L., Zhai P. (2009). The dynamic properties of SiCp/Al composites fabricated by spark plasma sintering with powders prepared by mechanical alloying process. Mater. Sci. Eng. A.

[22] Owolabi G.M., Odeshi A.G., Singh M.N.K., Bassim M.N. (2007). Dynamic shear band formation in Aluminum 6061-T6 and Aluminum 6061-T6/Al2O3 composites. Mater. Sci. Eng. A.

[23] Tang B., Wang H., Jin P., Jiang X. (2020). Constitutive flow behavior and microstructural evolution of 17 vol% SiCp/7055Al composite during compression at elevated temperature. J. Mater. Res. Technol..

[24] Wang K., Li X., Li Q., Shu G., Tang G. (2017). Hot deformation behavior and microstructural evolution of particulate-reinforced AA6061/B4C composite during compression at elevated temperature. Mater. Sci. Eng. A.

[25] Chen S., Teng J., Luo H., Wang Y., Zhang H. (2017). Hot deformation characteristics and mechanism of PM 8009Al/SiC particle reinforced composites. Mater. Sci. Eng. A.

[26] Wang Y.Z., Cavaliere P., Spigarelli S., Evangelista E. (2001). Temperature and strain-rate sensitivity parameters: Analysis of the deformed metal matrix composite A359/SiC/20p. J. Mater. Sci. Lett..

[27] Sun W., Duan C., Yin W. (2020). Development of a dynamic constitutive model with particle damage and thermal softening for Al/SiCp composites. Compos. Struct..

[28] Masuda T., Kobayashi T., Wang L., Toda H. (2003). Effects of Strain Rate on Deformation Behavior of A6061-T6 Aluminum Alloy. Mater. Sci. Forum.

[29] Lee W.-S., Sue W.-C., Lin C.-F., Wu C.-J. (2000). The strain rate and temperature dependence of the dynamic impact properties of 7075 aluminum alloy. J. Mater. Process. Technol..

[30] Djapic Oosterkamp L., Ivankovic A., Venizelos G. (2000). High strain rate properties of selected aluminium alloys. Mater. Sci. Eng. A.

[31] Suo Y., Deng Z., Wang B., Gong Y., Jia P. (2021). Constitutive model of metal matrix composites at high strain rates and its application. Mater. Today Commun..

[32] Li Y., Ramesh K.T., Chin E.S.C. (2000). Viscoplastic deformations and compressive damage in an A359/SiCp metal–matrix composite. Acta Metall. Mater..

